# Supplementary material for: Interplay between OmpA and RpoN Regulates Flagellar Synthesis in Stenotrophomonas maltophilia
Source: Microorganisms. 2021 Jun 4;9(6):1216. doi: 10.3390/microorganisms9061216 (PMC8229486; doi:10.3390/microorganisms9061216)
Supplement: Supplementary file 1 [file microorganisms-09-01216-s001.zip › microorganisms-1208150-supplementary.pdf]

Supplementary files

Interplay between OmpA and RpoN regulates flagellar synthesis in *Stenotrophomonas maltophilia*

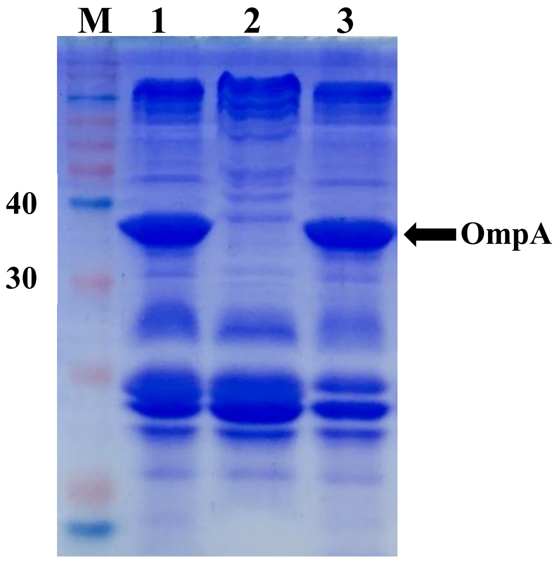

Figure S1. The SDS-PAGE of outer membrane proteins from wild-type KJ, KJΔOmpA, and KJL2::OmpAΔOmpA.

Outer membrane proteins were prepared by *N*-lauroylsarcosine method. Equal protein concentrations were separated by SDS-PAGE with a 5% stacking gel and a 15% separating gel, and stained with Coomassie brilliant blue. Lane M, molecular weight standards; lane 1, KJ; lane 2, KJΔOmpA; lane 3, KJL2:OmpAΔOmpA. The black arrow indicates OmpA protein

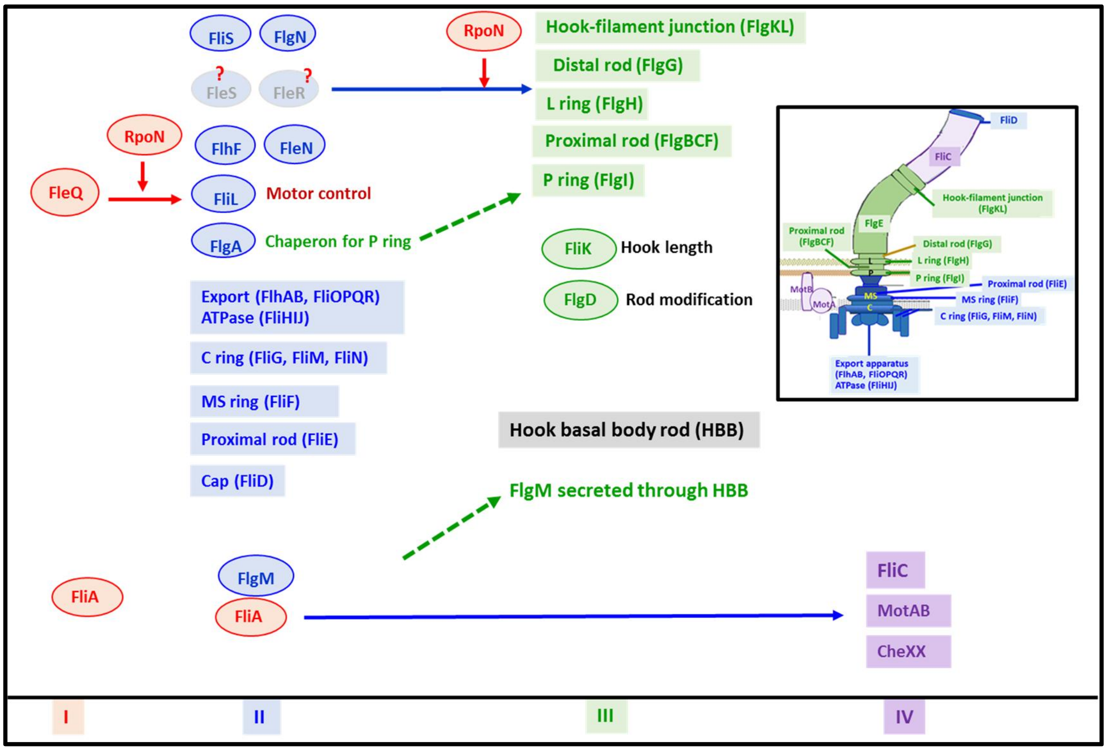

Figure S2. The preliminary flagellum synthesis model of *S. maltophilia* based on the known *P. aeruginosa* model. The genes classified in class I, II, III, and IV are marked in red, blue, green, and purple, respectively. The genes symbolized by ovals represent the encoded proteins are composed of flagellum and by rectangles are regulatory proteins.

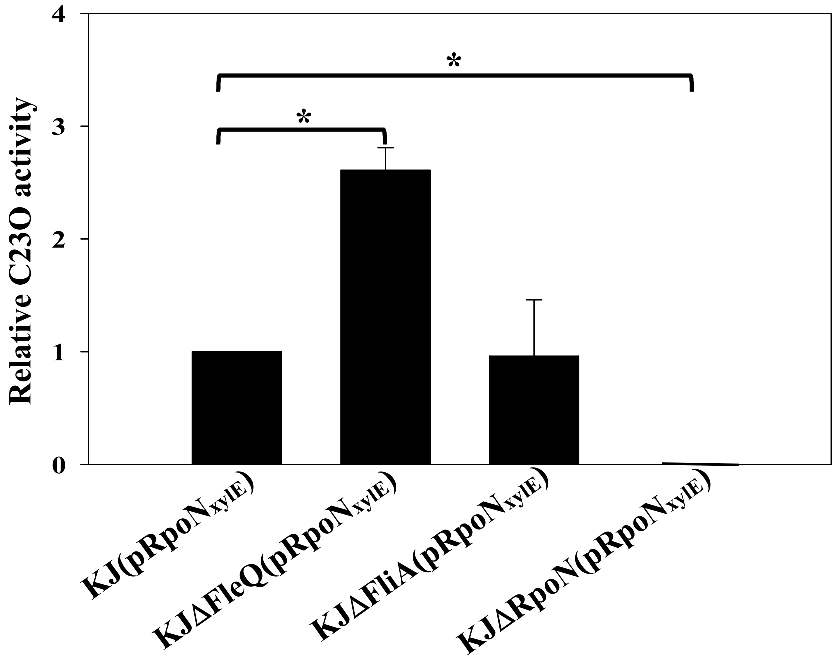

Figure S3. The C23O activities of KJ(pRponN$_{xylE}$), KJ(pRpoN$_{xylE}$), KJΔFleQ(pRpoN$_{xylE}$), KJΔFliA(pRpoN$_{xylE}$), and KJΔRpoN(pRpoN$_{xylE}$). Overnight cultures of bacteria cells tested were inoculated into fresh LB with an initial OD450nm of 0.15 and then cultured for 5 h. The C23O activities were measured. All values were normalized to the activity of KJ(pRpoN$_{xylE}$) cells. Data are the means from three independent experiments. Error bars indicate the standard deviations for three triplicate samples. *, $P < 0.05$, significance calculated by Student's *t* test.

Table S1 Bacterial strains, plasmids and primers used in this study

Strain, plasmid, or primer	Genotype or properties	Reference
S. maltophilia		
KJ	A clinical *S. maltophilia* isolate	1
KJΔOmpA	*S. maltophilia* KJ mutant of *ompA* gene; *ΔompA*	This study
KJΔFliC1C2C3	*S. maltophilia* KJ mutant of *fliC1, fliC2,* and *fliC3* genes; *ΔfliC1, ΔfliC2, ΔfliC3*	This study
KJΔRpoN	*S. maltophilia* KJ mutant of *rpoN* gene; *ΔrpoN*	This study
KJΔFleQ	*S. maltophilia* KJ mutant of *fleQ* gene; *ΔfleQ*	This study
KJΔFliA	*S. maltophilia* KJ mutant of *fliA* gene; *ΔfliA*	This study
KJL2::OmpA	The L2 gene of *S. maltophilia* KJ replaced with *ompA* gene; *L2::ompA*	This study
KJL2::RpoN	The L2 gene of *S. maltophilia* KJ replaced with *rpoN* gene; *L2::rpoN*	This study
E. coli		
DH5α	F- φ80d/*acZΔM15* Δ(*lacZYA-argF*)*U169 deoR recA1 endA1 hsdR17* (r_k^- m_k^+) *phoA supE44λ thi-1 gyrA96 relA1*	Invitrogen
S17-1	λ*pir*$^+$ mating strain	2
**Plasmids**		
pEX18Tc	*sacB oriT*, Tc^r	3
pRK415	Mobilizable broad-host-range plasmid cloning vector, RK2 origin; Tc^r	4
pRKxylE	pRK415-derived plasmid, carrying a complete *xylE* gene, whose orientation is opposite to P_{lacZ} of pRK415; Tc^r	5
pEXCJ1	pEX18Tc-derived plasmid, a 353-bp DNA upstream of *L2* gene inserted into HindIII and SphI sites, and a 376-bp DNA downstream of *L2* gene inserted into SacI and EcoRI sites; Tc^r	This study
pΔOmpA	pEX18Tc with an internal-deleted *ompA* gene; Tc^r	This study
pΔFliC1C2C3	pEX18Tc with deleted *fliC1, fliC2, and fliC3* genes; Tc^r	This study
pΔRpoN	pEX18Tc with an internal-deleted *rpoN* gene; Tc^r	This study
pΔFleQ	pEX18Tc with an internal-deleted *fleQ* gene; Tc^r	This study
pΔFliA	pEX18Tc with an internal-deleted *fliA* gene; Tc^r	This study
pCJ1-OmpA	pEXCJ1 with an intact *ompA* gene; Tc^r	This study
pCJ1-RpoN	pEXCJ1 with an intact *rpoN* gene; Tc^r	This study
$pRpoN_{xylE}$	pRK415 with a P_{rpoN}*::xylE* transcriptional fusion construct; Tc^r	This study

Primers

OmpAN-F	5’-GTAAGCTTGCAGACCACCTTCGAGA-3’	This study
OmpAN-R	5’-TGTCTAGAAGCGTCGGTACCCTTC-3’	This study
OmpAC-F	5’-GCGTCTAGAACCGTCGTACCGA-3’	This study
OmpAC-R	5’-CTGAGCTCCACGGATGTAGAGCT-3’	This study
FliC1N-F	5’-CTGAGCTTGTACCAGCTGCA-3’	This study
FliC1N-R	5’-GAGGATCCAGCGACATCGTA-3’	This study
FliC3C-F	5’-AAGCATGCCGCAGAACTGA-3’	This study
FliC3C-R	5’-CAAAGCTTGCCATGTGATTCT-3’	This study
RpoNN-F	5’-CGGGTACCCATGATGGAGTCA-3’	This study
RpoNN-R	5’-CGGGATCCGGTGAAGTTGTT-3’	This study
RpoNC-F	5’-AAGGATCCGAGGAAATCGGC-3’	This study
RpoNC-R	5’-AGTCTAGATTGCGGAAGTGG-3’	This study
FleQN-F	5’-CCTCTAGAAGGAACAGTCGT-3’	This study
FleQN-R	5’-GAGTCGACCCGCAGTTGAT-3’	This study
FleQC-F	5’-AGGTCGACAAGTACGGCAT-3’	This study
FleQC-R	5’-GGAAGCTTCCGATTGTGACCT-3’	This study
FliAN-F	5’-TCGGTACCGATGTCTCGCTGA-3’	This study
FliAN-R	5’-CCTCTAGAGGTCCGAATGCT-3’	This study
FliAC-F	5’-GGTCTAGAAGCGTGGTGAGT-3’	This study
FliAC-R	5’-CGAAGCTTAGGTTCTTGACGA-3’	This study
pEXCJ1N-F	5’-CCAAGCTTGAGACGATGGAACA-3’	This study
pEXCJ1N-R	5’-GAGCATGCGGGTTCTCCTGGGGGA-3’	This study
pEXCJ1C-F	5’-GTGAGCTCGGGCGAGGTACT-3’	This study
pEXCJ1C-R	5’-GGGAATTCCGTGTGGTTCGAA-3’	This study
OmpA-F	CTGCATGCACAAGAAGATCCT-3’	This study
OmpA-R	5’-GTTCTAGAGTGTCCTACTGGCA-3’	This study
RpoN-F	5’-GATCTAGAGCAAGATGCGTT-3’	This study
RpoN-R	5’-GCGGTACCGTAAGCTTGTT-3’	This study
FliC3his-F	5’-GCCATATGGCACAAGTCATCA-3’	This study
FliC3his-R	5’-CCGAATTCTTGCGCAGCAGG-3’	This study
RpoNQ109-F	5’-GGAAGTAGCCGAGGAAATCG-3’	This study
RpoNQ109-R	5’-GGCGAAGAAATGCTTCAGTT-3’	This study
FleQQ103-F	5’-CTGATCAACGAAGCACTGGA-3’	This study
FleQQ103-R	5’-CGTACTTGCGCAGCTTTTC-3’	This study
FliAQ117-F	5’-GCTGGAGCGTGGTGAGTT-3’	This study
FliAQ117-R	5’-CCGATCTCCTTCAGGTTCAG-3’	This study
FliD90Q-F	5’-CTCAAGGTCAAGGGCAAGAG-3’	This study
FliD90Q-R	5’-GTTGTAGGCGGTCACGAACT-3’	This study

FliNQ91-F	5’-GTGGTGGAACTGGAACGTG-3’(需 confirm)	This study
FliNQ91-R	5’-CATTGATCACCACCACTTCG-3’	This study
FlgGQ97-F	5’-CACCGCCAAGAATTTTGAAC-3’	This study
FlgGQ97-R	5’-AGGCATCTGAACCTCGAAGA-3’	This study
FlgKQ90-F	5’-CCAGAACATGCTGGATGGTG-3’	This study
FlgKQ90-R	5’-TTGTTGACCTCGCTGTTGAG-3’	This study
FliC3Q109-F	5’-CAATACGATGTCGCTCAACG-3’	This study
FliC3Q109-R	5’-GTCCTTCGCGCTGTTGAT-3’	This study
CheVQ108-F	5’- CTCGATCCAGGGCTTCCT-3’	This study
CheVQ108-R	5’-GCGGTCAGGTAGCTCGATT-3’	This study
MotCQ109-F	5’-CAGGAGCACCAGGACCAG-3’	This study
MotCQ109-R	5’-TCTTCATCACCGCGATCAG-3’	This study
FliJQ93-F	5’-GAGTACGCCAACGCTCAGAT-3’	This study
FliJQ93-R	5’-CTGTTCAACGGCACTGTCC-3’	This study
FliLQ107-F	5’-CCTTCGTGGTCAACCTCAAC-3’	This study
FliLQ107-R	5’-ATGGGTCTTGATCGCTTCC-3’	This study
FliEQ90-F	5’-CAACGATGCGCAACAGAA-3’	This study
FliEQ90-R	5’-GAGGCGACCATCACCTTG-3’	This study
FliKQ90-F	5’-CGACCAGAAAATCGGACAC-3’	This study
FliKQ90-R	5’-ACCTTGTCGCCGTTCAACT-3’	This study
FlgBQ90-F	5’-AGGACCTGGACTTCGATGC-3’	This study
FlgBQ90-R	5’-TTTCGTAGTGCTGCTCATGG-3’	This study
16rDNAQ-F	5’- GACCTTGCGCGATTGAATG -3’	6
16rDNAQ-R	5'- CGGATCGTCGCCTTGGT -3’	6

References

1. Hu RM, Huang KJ, Wu LT *et al*. Induction of L1 and L2 β-lactamases of *Stenotrophomonas maltophilia*. *Antimicrob Agents Chemother* 2008; **52**:1198-200.
2. Simon R, O'Connell M, Labes M *et al*. Plasmid vector for the genetic analysis and manipulation of *Rhizobia* and other Gram-negative bacteria. *Methods Enzymol* 1986; **118:** 640-59.
3. Hoang TT, Karkhoff-Schweizer RR, Kutchma AJ *et al*. A broad-host-range Flp-FRT recombination system for site-specific excision of chromosomally-located DNA sequences: application for isolation of unmarked *Pseudomonas aeruginosa* mutants. *Gene* 1998; **212**:77-86.
4. Keen NT, Tamaki S, Kobaysahi D *et al*. Improved broad host-range plasmids for DNA cloning in gram-negative bacteria. *Gene* 1988; **70**:191-7.
5. Huang YW, Liou RS, Lin YT *et al*. A linkage between SmeIJK efflux pump, cell envelope integrity, and σE-mediated envelope stress response in *Stenotrophomonas maltophilia*. *PLoS One* 2014; **9**: e111784.

6. Chen CH, Huang CC, Chung TC *et al*. Contribution of resistance-nodulation-division efflux pump operon *smeU1-V-W-U2-X* to multidrug resistance of *Stenotrophomonas maltophilia*. *Antimicrob Agents Chemother* 2011; **55:** 5826-33.

Table S2. Comparison of the flagella synthesis-associated proteins between *S. maltophilia* and *P. aeruginosa*

S. maltophilia			***P. aeruginosa***		**Protein identity(%)**
**Smlt**	**Protein**	**Protein function**	**PAO**	**Protein**	**/similarity(%)**
2295	FleQ	transcriptional activator	1097	FleQ	44%/61%
2296	FleX	transcriptional regulator			
2297	RpoN	σ54 sigma factor	4462	RpoN	39%/55%
2270	FliA	σ28 sigma factor	1455	FliA	47%/64%
2271	FleN	flagella number regulator	1454	FleN	52%/70%
2272	FlhF	flagellar polar location	1453	FlhF	29%/42%
2273	FlhA	flagellar export protein	1452	FlhA	59%/75%
2274	FlhB	flagellar export protein	1449	FlhB	42%/60%
2277	FliR	flagellar export protein	1448	FliR	36%/54%
2278	FliQ	flagellar export protein	1447	FliQ	40%/59%
2279	FliP	flagellar export protein	1446	FliP	54%/67%
2280	FliO	flagellar export protein	1445	FliO	35%/51%
2281	FliN	flagellar motor switch protein	1444	FliN	48%/60%
2282	FliM	flagellar motor switch protein	1443	FliM	53%/72%
2283	FliL	basal body-associated protein	1442	FliL	35%/53%
2285	FliJ	chaperone, export of hook proteins	1105	FliJ	27%/46%
2286	FliI	flagellum-specific ATPase	1104	FliI	55%/70%
2287	FliH	flagella assembly protein	1103	FliH	22%/37%
2288	FliG	motor switch protein	1102	FliG	57%/79%
2289	FliF	Basal body MS ring	1101	FliF	38%/55%
2290	FliE	Basal body MS ring/rod adapter	1100	FliE	39%/56%
2302	FliS	chaperone for filament elongation	1095	FliS	41%/57%
2303	FliD	filament cap	1094	FliD	29%/46%
2319	FlgA	basal body P-ring protein	3350	FlgA	25%/43%
2320	FlgM	anti-sigma factor FlgM	3351	FlgM	26%/53%
2321	FlgN	chaperone	3352	FlgN	23%/39%
		hypothetical protein	1096	FleP	
		TCS-sensor kinase	1098	FleS	
		TCS-response regulator	1099	FleR	

2284	FliK	flagellar hook-length control	1441	FliK	28%42%
2307	FlgL	hook-filament junctional protein	1087	FlgL	27%/47%
2308	FlgK	hook-filament junctional protein	1086	FlgK	34%/50%
2309	FlgJ	flagellum specific muramidase	1085	FlgJ	32%/45%
2310	FlgI	basal body P-ring	1084	FlgI	60%/73%
2311	FlgH	basal body L-ring	1083	FlgH	46%/58%
2312	FlgG	basal body rod	1082	FlgG	59%/73%
2313	FlgF	basal body rod	1081	FlgF	37%/56%
2314	FlgE	hook	1080	FlgE	41%/56%
2315	FlgD	hook capping protein	1079	FlgD	34%/55%
2316	FlgC	basal body rod	1078	FlgC	38%/60%
2317	FlgB	basal body rod	1077	FlgB	40%/60%
2265	MotB	flagellar motor protein	1461	MotD	37%/52%
2266	MotC	flagellar motor protein	1460	MotC	56%/72%
2267	CheA	chemotaxis sensor kinase regulator	1458	CheA	45%/57%
2268	CheZ	chemotaxis protein	1457	CheZ	30%/41%
2269	CheY	chemotaxis response regulator	1456	CheY	72%/84%
2304	FliC1	flagellin	1092	FliC	42%/53%
2305	FliC2	flagellin	1092	FliC	40%/53%
2306	FliC3	flagellin	1092	FliC	40%/54%
2318	CheV	chemotaxis response regulator			
